# The Effect of Using a Normothermia Checklist on Awakening Time From Anesthesia and Coagulation Disorder: A Randomized Controlled Trial

**DOI:** 10.1097/jnr.0000000000000583

**Published:** 2023-11-24

**Authors:** Pinar YILMAZ EKER, Meryem YILMAZ

**Affiliations:** 1 PhD, RN, Assistant Professor, Department of Nursing, Health College, Sivas Cumhuriyet University, Sivas, Turkey; 2 PhD, RN, Professor, Faculty of Health Sciences, Department of Nursing, Sivas Cumhuriyet University, Sivas, Turkey.

**Keywords:** inadvertent perioperative hypothermia, checklist, coagulation disorder, awaken from anesthesia, nursing

## Abstract

**Background:**

Inadvertent perioperative hypothermia (IPH) is a common issue in surgical patients. To avoid this issue, the patient should be monitored continuously throughout the perioperative process. Evidence-based practices in line with relevant guidelines are necessary to maintain normothermia.

**Purpose:**

This study was developed to determine the effect of using a control list developed for preventing IPH on time of awakening from anesthesia and coagulation disorder in surgical patients.

**Methods:**

In this randomized controlled study, nursing interventions were applied to patients in accordance with the normothermia checklist (NC) developed by the researchers to prevent IPH.

**Results:**

In this study, 30 patients were respectively assigned to the experimental and control groups. Conducting nursing interventions in accordance with the control checklist was found to be effective in preventing IPH. Moreover, time of awakening from anesthesia was significantly shorter in the experimental group (3.77 ± 1.10 minutes) than the control group (11.03 ± 2.51 minutes; *p* < .05). Furthermore, tendency to bleed was higher in the control group than the experimental group, and a statistically significant between-group difference in coagulation disorders was found (*p* < .05).

**Conclusions/Implications for Practice:**

The results of this evidence-based study indicate that implementing nursing interventions in line with the developed NC is effective in preventing IPH. Preventing IPH, which increases the risk of numerous complications in surgical patients, is an important responsibility of nurses. Nurses may employ the NC proposed in this study to better secure the safety and minimize the risk of complications in surgical patients.

## Introduction

Inadvertent perioperative hypothermia (IPH) remains a clinical challenge for nurses caring for patients undergoing surgery (Russell et al., 2022). IPH is an identifiable and preventable condition (Ralph et al., 2020). All members of the surgical team, especially surgical nurses, have important roles with regard to preventing IPH throughout the perioperative period. The primary priority of nurses in preventing IPH is to treat symptoms; warm patients appropriately; and monitor patients before, during, and after surgery (De Mattia et al., 2013). Studies in the literature (Giuliano & Hendricks, 2017; Munday et al., 2019; Tamer & Karadağ, 2020) have reported insufficient awareness of IPH, lack of clarity regarding the definitions of hypothermia and normothermia, and a need to increase knowledge related to IPH management among nurses.

To effectively lead the perioperative team in interventions to prevent IPH, nurses should be aware of the demographic (e.g., age, gender) and operative (e.g., type of surgery, duration) factors that elevate the risk of IPH in patients. Identifying at-risk patients facilitates informed decision making regarding the frequency and method of temperature monitoring and determines what interventions (e.g., warm blankets, heating devices) should be used to help these patients maintain normothermia (Akers et al., 2019). Patients have the right to receive the highest possible quality of care, and nurses have a professional responsibility to ensure that patients receive this care (Healy et al., 2019).

Appropriate guidelines may be used to help prevent IPH and maintain normothermia. Clinical practice guidelines are systematically developed recommendations or statements designed to assist the practitioner and/or patient to make appropriate health decisions in specific clinical situations. Care based on evidence-based clinical practice guidelines has been recognized as a key component in improving the quality, safety, efficacy, and effectiveness of healthcare and has been reported to positively impact patient outcomes (Yang et al., 2022). Many guidelines have been developed in Turkey and around the world to prevent IPH in surgical patients. The American Society of PeriAnesthesia Nurses (2021), the National Institute of Clinical Evidence (2021), and the Association of periOperative Registered Nurses (2016) have published material that outlines various practices for maintaining normothermia throughout the perioperative period. In Turkey, the “Guidelines for the Prevention of Inadvertent Perioperative Hypothermia” was published in 2013 by the Turkish Society of Anesthesiology and Reanimation (2013), providing evidence on the subject and including application recommendations similar to other guidelines. However, this evidence is not currently used in healthcare institutions. Therefore, to prevent complications, it is important to establish and implement applicable protocols with proven effectiveness to guide healthcare professionals to prevent IPH. Therefore, this study was carried out to determine the effect of using the normothermia checklist (NC) developed for the prevention of IPH in surgical patients on time of awakening from anesthesia and coagulation disorder. This research was conducted to support the principle that the standardization of surgical nursing care increases quality of care. This is the only study known to the authors designed to evaluate recovery time from anesthesia in the nursing field in Turkey.

## Methods

### Design, Participant, and Setting

In this randomized, controlled, prospective, experimental case–control study, data were collected in the general surgery clinic and operating room of Sivas Cumhuriyet University Research and Practice Hospital between October 10, 2019, and August 10, 2020. Patients who had undergone surgical intervention in the General Surgery Clinic of Sivas Cumhuriyet University Research and Practice Hospital, who met the inclusion criteria, and who agreed to participate were included in the study. The inclusion criteria were as follows: (a) elective surgery patients aged 18–65 years, (b) having an American Society of Anesthesiologists (ASA) score of I–II, and (c) being hospitalized for at least 24 hours after surgery. The exclusion criteria were as follows: (a) having a neurological, psychiatric, or neuromuscular disease; (b) being addicted to alcohol and/or drugs; (c) using medications that affect thermoregulation such as vasodilators; (d) having a history of thyroid disease; (e) having a cancer diagnosis; (f) being or suspected of being pregnant; and (g) having a fever and infection.

The sample size of the study was determined using the G*Power 3.1.9.7 program. In the power analysis, with *α =* .05, β = 0.10, 1 − β = 0.90, and effect size = 0.8, 30 individuals were respectively required for the experimental and control groups, with a power of 0.91 achieved. During the study period, patients who met the sampling inclusion criteria and were admitted to the clinic for elective surgery were stratified by gender and type of surgery and then randomized into blocks. Using a random-number table, the first patient was assigned to the experimental group, and subsequent patients were sampled until the layers and blocks were equalized in both groups. The study was completed with 60 patients, including 30 in the experimental group and 30 in the control group. To confirm the intergroup homogeneity after randomization, the two groups were compared using a chi-square test, with the results showing no statistically significant between-group difference (Table 1).

**Table 1.** Comparison of Experimental and Control Groups in Terms of Demographic and Surgical Characteristics (*N* = 60)

Descriptive Characteristic	Experimental Group		Control Group		χ^2^	*p*
	*n*	%	*n*	%		
Age (years)					0.30	.584
18–40	9	30.0	11	36.7		
41–65	21	70.0	19	63.3		
Body mass index (kg/m^2^)					0.07	.787
≤ 25	11	36.7	10	33.3		
> 25	19	63.3	20	66.7		
Gender					1.09	.297
Female	15	50.0	19	63.3		
Male	15	50.0	11	36.7		
Diagnosis					0.00	1.000
Laparoscopic cholecystectomy	21	70.0	21	70.0		
Bariatric surgery (sleeve gastrectomy)	3	10.0	3	10.0		
Right inguinal hernia	3	10.0	3	10.0		
Bilateral inguinal hernia	1	3.3	1	3.3		
Hiatal hernia	1	3.3	1	3.3		
Laparoscopic cholecystectomy + umbilical hernia	1	3.3	1	3.3		
Chronic disease					0.08	.774
Yes	9	30.0	8	26.7		
No	21	70.0	22	73.3		
Type of chronic disease					4.94	.176
Diabetes mellitus	1	11.1	1	14.3		
Hypertension	5	55.6	4	57.1		
Asthma/chronic obstructive pulmonary disease	0	0.0	2	28.6		
Diabetes mellitus + Hypertension	3	33.3	0	0.0		
ASA score					0.34	.559
ASA I	21	70.0	23	76.7		
ASA II	9	30.0	7	23.3		
Premedication					0.00	1.000
Yes	1	3.3	1	3.3		
No	29	96.7	29	96.7		
Surgery time (minutes)					0.16	.921
30–60	3	10.0	3	10.0		
61–120	23	76.7	24	80.0		
> 120	4	13.3	3	10.0		
Development of hypothermia					6.00	< .001**
Yes	0	0.0	30	100.0		
No	30	100.0	0	0.0		
Hypothermia level						
Mild (34°C–36°C)	0	0.0	30	100.0		

	*M*	*SD*	*M*	*SD*	*t*	*p*
Age (years)	43.73	11.18	44.60	8.78	0.33	.740
Body mass index	30.07	8.36	30.16	6.27	0.08	.963

*Note.* ASA = American Society of Anesthesiologists.

**p < .001.

### Instrument and Procedure

Two data collection tools were used in the study.

#### Questionnaire form

The two-part questionnaire was prepared after referencing the hypothermia risk factors cited in the literature (Collins et al., 2019; Healy et al., 2019). The first part included nine questions gathering information on the respondent's age, gender, diagnosis, body mass index (BMI), chronic disease history, ASA score, premedication status, and operation time. The second part collected data on time of awakening from anesthesia and presurgery and postsurgery laboratory findings related to coagulation status.

#### Normothermia checklist

The NC, comprising five parts, was created in line with guidelines developed by the Association of periOperative Registered Nurses, American Society of PeriAnesthesia Nurses, National Institute of Clinical Evidence, and Turkish Society of Anesthesiology and Reanimation. The NC included nursing interventions used to prevent IPH and maintain normothermia that should be applied for 24 hours during the preoperative clinic, preparation unit, operating room, postoperative anesthesia care unit (PACU), and postoperative clinic periods. The original version of the checklist may be obtained from the corresponding author. The interventions included in the NC are detailed below:

##### 1. In the preoperative clinic

The temperature of the patient's room is measured with a thermometer. The patient's body temperature (BT) is measured temporarily. If the BT is below 36°C, active heating is provided, and if it is above 36°C, passive isolation methods are used to maintain normothermia. Patients with BT below 36°C are monitored at 15-minute intervals, whereas those with BT above 36°C are monitored at 4-hour intervals. After achieving normothermia, patients are sent to the operating room.

##### 2. In the preparation room

Patients are sent from the clinic to the preparation room at least 20 minutes before surgery. The temperature of the preparation room is measured with a thermometer, and the patient's BT is measured temporarily. If the BT is below 36°C, active heating is provided, and if it is above 36°C, passive isolation methods are used to maintain normothermia. Patients with BT above 36°C are taken to the operating room.

##### 3. In the operating room

The operating room temperature is measured with a thermometer. The patient's BT is measured temporarily. If it is above 36°C, anesthesia induction is started, and if it is below 36°C, anesthesia induction is delayed until normothermia is achieved. If the intravenous (IV) fluids, blood, and blood products to be administered to the patient exceeds 1,000 ml, all irrigation fluids are heated to 38°C–40°C. At-risk patients are actively warmed, even if the intervention takes less than 30 minutes. The BT of the patient is measured from the lower end of the esophagus in the operating room and is monitored temporarily at 15-minute intervals.

##### 4. In the postoperative anesthesia care unit

PACU temperature is measured with a thermometer, and the patient's BT is measured temporarily. If the BT is above 36°C and other transfer conditions are met (e.g., conscious, other vital signs are normal), they are transferred to the clinic. If the BT is below 36°C, patients are transferred to the clinic after achieving normothermia with an active warming method.

##### 5. In the postoperative clinic (within the first 24 hours)

The temperature of the patient's room is measured with a thermometer, and BT is measured temporarily. When the patient comes to the clinic, their clothes are put on. The BT of the patient is measured every 15 minutes in the first hour, every 30 minutes in the second hour, and at 1-hour intervals thereafter. If the BT is below 36°C, active heating is provided, and if it is above 36°C, passive isolation methods are used to maintain normothermia. Patients with BT below 36°C are monitored at 15-minute intervals, whereas those with BT above 36°C are monitored at 4-hour intervals.

The details of this study were explained to the participants, and written consent was obtained. The patients in the experimental and control groups were composed of individuals with similar characteristics in terms of medical diagnosis and gender. After applying interventions in line with the checklist, the participants were evaluated in terms of coagulation disorder status and degree of difficulty experienced in awakening from anesthesia. The NC form was applied only to the experimental group. The experimental group having a lower rate of coagulation disorders and less difficulty in awakening from anesthesia was defined as the indicator of NC effectiveness. The suitability of NC applied by the researcher for surgical patients and its applicability to institutions were also evaluated. In this study, blood was only taken from the patients in line with routine practice, and test results available in the patient's file (prothrombin time [PT], activated partial thromboplastin time [aPTT], international normalized ratio [INR], platelet [PLT], hematocrit [HCT], hemoglobin [Hg], serum calcium [Ca^+2^]) were evaluated and recorded. Time of awakening from anesthesia was measured in minutes after administering Bridion 200 mg/2 ml (active ingredient: sugammadex) IV to ensure standardization, terminate the anesthetic gas given to the patient, and restore the patient's muscle functions. A temporal temperature-measuring device was used to monitor the patient's BT during data collection. The literature lacks consensus regarding the optimal temperature-measuring device for patients (Erdling & Johansson, 2015; Gabriel et al., 2019). The usability and accuracy of the device are important for its suitability for regular use. In addition, it should be possible to use it for the entire perioperative period, which covers before, during, and after surgery (Gabriel et al., 2019). Therefore, in this study, a temporal thermometer device was used because it is portable, has no calibration problems, and does not pose a problem in terms of patient comfort.

Experimental group participants were warmed using the active heating technique with a forced-air warming system and a forced-air warming blanket. A heating device was used concurrently to heat IV fluids and irrigation fluids. In this study, IPH prevention standards were not implemented because of a lack of sufficient materials and health workers in the study hospital. Participants in the control group received standard care involving passive isolation methods; these methods encompass the patient being covered with blankets in the PACU, being allowed to dress in their own clothes and socks after transfer to the patient room, and being covered with a blanket. A portable room thermometer was used to measure the ambient temperature for both groups. All of the patients were anesthetized intravenously and administered the same drugs based on their weight as measured in kilograms. The drugs that were administered to the patients included a Pentothal vial (active ingredient: sodium thiopental) 4 mg/kg, a fentanyl citrate ampoule (active ingredient: fentanyl) 2 mcg/kg, and a brown vial (active ingredient: rocuronium bromide) 0.1 mg/kg. All of the procedures on the NC checklist were made by the first author of the study. To ensure that measurements were unbiased, the records were taken by the anesthesia technician, who was a member of the surgical team.

### Ethical Considerations

Before starting the study, permission was obtained from the Sivas Cumhuriyet University Clinical Research Ethics Committee (93596471-010.99-E.30220).

### Statistical Analysis

Statistical analysis was performed using SPSS for Windows 22.0 (IBM Inc., Armonk, NY, USA). The descriptive statistics were analyzed, and the number and percentage distribution were determined. In terms of skewness and kurtosis, the scores for all of the variables except for the minimum intraoperative BT variable were found to be insignificant, with *p* > .05. As a result, parametric analysis was applied to the data. Chi-square test and *t* test were used on numerical data to assess differences between the experimental and control groups in terms of descriptive properties. The chi-square test and Mann–Whitney *U* test were used to determine the developmental status of IPH between the groups and the risk factors for IPH in the control group. Logistic regression analysis was used to determine the probabilities (risk coefficients) of risk factors in the development of IPH, with the level of significance defined as *p* < .05.

### Research Hypotheses

The following are this study's hypotheses:

H0_a_: Interventions following the NC do not positively affect time of awakening from anesthesia.
H0_b_: Interventions following the NC do not positively affect risk of coagulation disorder.
H1_a_: Interventions following the NC positively affect time of awakening from anesthesia.
H1_b_: Interventions following the NC positively affect risk of coagulation disorder.

## Results

### Participant Demographics

Demographic and surgical characteristics in the experimental and control groups are compared in Table 1. Seventy percent of the experimental group and 63.3% of the control group were aged 41–65 years. The mean age was 43.73 (*SD* = 11.18) years in the experimental group and 44.60 (*SD* = 8.78) years in the control group, with a statistically insignificant difference (*t* = .33, *p* = .740). Half (50%) of the experimental group and 63.3% of the control group were female. Between-group differences in demographic data were insignificant with the exception of hypothermia development (*p* > .05). None in the experimental group developed IPH, and 100% of the controls developed IPH, which was a statistically significant difference (χ^2^ = 6.00, *p* < .001). These findings indicate a difference between the experimental and control groups in terms of developing hypothermia, with all in the control group developing mild hypothermia (34°C–36°C).

### Time of Awakening From Anesthesia

In terms of mean time of awakening from anesthesia, the experimental group took 3.77 (*SD* = 1.10) minutes and the control group took 11.03 (*SD* = 2.51) minutes, indicating a statistically significant difference (*t* = 14.51, *p* < .001) in favor of the experimental group.

### Laboratory Test Information

Changes in blood values before and after the operation for both groups are compared in Table 2. No significant between-group difference was found in terms of PT, aPTT, INR, PLT, HCT, Hg, and Ca^+2^ values during the preoperative period. In the postoperative period, no significant between-group difference was found in terms of PT, aPTT, PLT, and HCT, and a significant between-group difference (*p* < .05) was found in terms of INR, Hg, and Ca^+2^. Thus, a difference in favor of the experimental group in terms of INR, Hg, and Ca^+2^ was found after surgery. The differences between preoperative and postoperative PT and aPTT values in the experimental group were statistically insignificant (*p* > .05). However, the differences between preoperative and postoperative INR, PLT, HCT, Hg, and Ca^+2^ values in the experimental group were statistically significant (*p* < .05), showing a difference in favor of the posttest based on the PLT values and against the posttest based on the preoperative and postoperative INR, HCT, Hg, and Ca^+2^ values in the experimental group. The differences between preoperative and postoperative PT, aPTT, INR, PLT, HCT, Hg, and Ca^+2^ values in the control group were statistically significant (*p* < .05). These findings show that the control group differed from the posttest in terms of preoperative and postoperative PT, aPTT, INR, PLT, HCT, Hg, and Ca^+2^ values.

**Table 2.** The Comparison of Experimental and Control Groups in Terms of Changes in Laboratory Findings Related to Preoperative and Postoperative Coagulation (*N* = 60)

Laboratory Tests Related to Coagulation	Pre-op						Post-op						EG		CG	
	EG		CG		*t*	*p*	EG		CG		*t*	*p*	Pretest–Posttest		Pretest–Posttest	
	*M*	*SD*	*M*	*SD*			*M*	*SD*	*M*	*SD*			*t*	*p*	*t*	*p*
PT (sec.)	12.20	0.72	11.97		0.79	1.20	.235	12.40	0.93	12.50	0.95	−0.43	.672	−1.74	.092	−3.36	.002*
aPTT (sec.)	25.57	4.44	27.47		5.07	−1.55	.127	26.52	3.86	28.29	5.47	−1.45	.153	−1.49	.147	−2.27	.031*
INR	1.05	0.06	1.04		0.06	0.67	.504	1.13	0.07	1.26	0.08	6.60	< .001**	−6.20	< .001**	−13.03	< .001**
PLT (1000/μL)	268.81	54.26	290.17		86.26	−1.15	.255	249.30	55.57	242.17	66.23	0.45	.653	7.58	< .001**	5.44	< .001*
HCT (%)	43.28	4.90	42.69		4.47	0.48	.632	40.52	4.65	38.04	5.86	1.82	.074	6.81	< .001**	7.35	< .001**	
Hg (g/dL)	14.38	1.71	14.03		1.85	0.78	.441	13.85	2.08	12.29	1.93	3.01	.004*	2.38	.024*	14.16	< .001**	
Ca^+2^ (mg/dL)	9.31	0.40	9.11		0.49	1.66	.103	8.90	0.40	8.15	0.53	6.12	< .001**	5.93	< .001**	12.16	< .001**

*Note.* Pre-op = preoperative laboratory findings; Post-op = postoperative laboratory findings; EG = experimental group; CG = control group; PT = prothrombin time; aPTT = activated partial thromboplastin time; INR = international normalized ratio; PLT = platelet; HCT = hematocrit; Hg = hemoglobin; Ca^+2^ = serum calcium.

*p < .05. **p < .001.

### Information of Hypothermia of Participants by Descriptive Characteristics

Hypothermia development status is compared based on participant introductory characteristics in Table 3. Two thirds (63.3%) of the participants who developed IPH and 70% of those who did not develop IPH were 41–65 years old. The difference between the groups (χ^2^ = 0.30, *p* = .584) was found to be statistically insignificant. According to the risk ratio, the rate of IPH development was 1.2 times higher in those aged 18–40 years. The BMI of 66.7% of the participants who developed IPH and 63.3% of those who did not develop IPH was > 25 kg/m^2^. The difference between the groups was found to be statistically insignificant (χ^2^ = 0.07, *p* = .787). These findings indicate that BMI is not a predictor of IPH development risk, with the risk of IPH development 1.1 times higher in the participants with BMI > 25 kg/m^2^. Two thirds (63.3%) of the participants who developed IPH and 50% of those who did not develop IPH were women. The gender characteristic was found to be statistically insignificant in between-group differences (χ^2^ = 1.09, *p* = .297), with the risk of IPH development in women 1.3 times higher than in men. The difference between the diagnoses of participants with and without IPH was statistically insignificant (χ^2^ = 0.00, *p* = 1.000). Three quarters (73.3%) of the participants who developed IPH and 70% of those who did not develop IPH had no chronic disease, which is a statistically insignificant difference (χ^2^ = 0.08, *p* = .774). The risk of developing IPH in individuals with chronic disease was 1 time higher compared with those without. Thus, the difference between the participants who developed IPH and those who did not develop IPH in terms of chronic disease types was statistically insignificant (χ^2^ = 4.94, *p* = .176). Three quarters (76.7%) of the participants who developed IPH and 70% of those who did not develop IPH were in the ASA I class, indicating a statistically insignificant between-group difference (χ^2^ = 0.34, *p* = .559). Thus, the risk of IPH development was 1.1 times higher in those with ASA I. The difference between patients who developed IPH and those who did not develop IPH in terms of premedication status was found to be statistically (χ^2^ = 0.00, *p* = 1.000) insignificant. These findings indicate that premedication status is not a predictor of IPH development risk.

**Table 3.** Comparison of Patients in Terms of Development of Hypothermia, by Descriptive Characteristics (*N* = 60)

Descriptive Characteristic	Hypothermia Development Status				χ^2^	*p*	Odds Ratio
	Developed		Not Developed				
	*n*	%	*n*	%			
Age (years)					0.30	.584	1.22
18–40	11	36.7	9	30.0			
41–65	19	63.3	21	70.0			
Body mass index (kg/m^2^)					0.07	.787	1.05
≤ 25	10	33.3	11	36.7			
> 25	20	66.7	19	63.3			
Gender					1.09	.297	1.27
Female	19	63.3	15	50.0			
Male	11	36.7	15	50.0			
Diagnosis					0.00	1.000	–
Laparoscopic cholecystectomy	21	70.0	21	70.0			
Bariatric surgery (sleeve gastrectomy)	3	10.0	3	10.0			
Right inguinal hernia	3	10.0	3	10.0			
Bilateral inguinal hernia	1	3.3	1	3.3			
Hiatal hernia	1	3.3	1	3.3			
Laparoscopic cholecystectomy + umbilical hernia	1	3.3	1	3.3			
Chronic disease					0.08	.774	.89
Yes	8	26.7	9	30.0			
No	22	73.3	21	70.0			
Type of chronic disease					4.94	.176	–
Diabetes mellitus	1	14.3	1	11.1			
Hypertension	4	57.1	5	55.6			
Asthma/chronic obstructive pulmonary disease	2	28.6	0	0.0			
Diabetes mellitus + hypertension	0	0.0	3	33.3			
ASA score					0.34	.559	1.10
ASA I	23	76.7	21	70.0			
ASA II	7	23.3	9	30.0			
Premedication					0.00	1.000	–
Yes	1	3.3	1	3.3			
No	29	96.7	29	96.7			

*Note.* ASA = American Society of Anesthesiologists.

### Perioperative Body Temperature

The preoperative, intraoperative, and postoperative BT in the experimental and control groups are compared in Table 4. Between-group differences in terms of preoperative minimum and maximum BT were statistically insignificant (*p* > .05). No between-group difference was found in terms of presurgery minimum and maximum BT. The differences between the minimum and maximum BT during surgery for the experimental and control groups were statistically significant (*p* < .05), showing a difference between the groups in terms of minimum and maximum BT during surgery. The mean minimum BT during surgery for the experimental group was higher than that of the control group. Statistically significant differences in postoperative minimum and maximum BT variations were found between the groups (*p* < .05). In addition, the results show the average minimum and maximum BT values for the experimental group after surgery to be higher than those for the control group.

**Table 4.** Comparison of Perioperative Body Temperature in the Experimental and Control Groups (*N* = 60)

Body Temperature in Perioperative Period	Experimental Group		Control Group		*t*	*p*
	*M*	*SD*	*M*	*SD*		
Preoperative (°C)						
Minimum	36.34	0.23	36.31	0.30	0.39	.702
Maximum	36.63	0.24	36.58	0.30	0.80	.425
Intraoperative (°C)						
Minimum	36.21	0.17	35.09	1.84	3.33	.002*
Maximum	36.70	0.18	36.07	0.20	12.95	< .001**
Postoperative (°C)						
Minimum	36.18	0.10	35.56	0.36	8.87	< .001**
Maximum	36.76	0.17	36.60	0.25	2.94	.005*

*p < .05. **p < .001.

## Discussion

Evidence-based care is provided to patients in line with guideline recommendations to increase patient and healthcare professional satisfaction because of improved patient outcomes. In this study, IPH did not develop during the perioperative period in those participants who were provided care in accordance with the evidence-based NC intervention developed by the researchers. Experimental group participants maintained normothermia from the moment they entered the preparation room until they left the PACU. However, control group participants maintained normothermia in the preparation room but experienced drops in their BT in the operating room. This finding provides evidence that the NC-based intervention developed by the researchers is beneficial to maintaining normothermia.

During the perioperative process, nurses can identify risk factors that may lead to IPH and provide nursing care to prevent adverse outcomes. Nurses should also participate in IPH prevention research, closely monitor patients for IPH during practices, and take warming measures to prevent IPH (Öner Cengiz et al., 2021). In some studies in which passive isolation or active warming techniques were used to prevent IPH in surgical patients (Koëter et al., 2013; McSwain et al., 2015; Williams, 2018), these techniques were found to have no effect on BT. However, other studies that used active warming techniques (Brodshaug et al., 2019; Granum et al., 2019; Rosenkilde et al., 2017) found active warming techniques to be more effective in preventing IPH before and after general anesthesia. In this study, the participants who were actively warmed did not develop IPH. To help clarify current questions about active warming in the literature, the experimental group in this study was given active heating methods such as fluid heaters and compressed air heating systems as well as warming using the passive insulation method, immediate dressing in the patient's own clothes, covering the patient with blankets and bedding, and keeping the ambient temperature optimal (patient room) in line with the NC guidelines. Interventions such as keeping the preparation room and PACU at 22°C–24°C and the operating room at > 21°C were used simultaneously. The positive results support that using active heating techniques and passive insulation together increases IPH prevention effectiveness. The passive isolation methods (dressing the patient, covering them with a blanket or bedding) applied during standard care to the control group were not adequate to prevent IPH. The combination of active warming and passive insulation methods applied in this study in accordance with NC guidelines was effective in preventing IPH in 100% of the control group. Although no similar studies have been conducted in Turkey, a recent Turkish study (Urfalioglu et al., 2021) reported active heating methods to have positive effects on IPH. Another study (Tunc et al., 2022) that compared active heating and passive insulation methods determined active heating to be effective in preventing IPH. Although the guidelines used to create the NC intervention used in this study apply both active and passive methods, related evidence on this issue remains inadequate.

In this study, it was determined that the use of NC significantly affected the time of awakening from anesthesia and prevention of IPH onset. Anesthesia was applied to all of the patients via IV using the same drugs, doses of which were adjusted based on participant BMI. Decreased BT may decrease cardiac output, and rhythm disturbances may occur. Low cardiac output slows the excretion of anesthesia from the body by negatively affecting circulation and drug pharmacokinetics (Shaikh & Lakshmi, 2014), which may delay patient awakening from anesthesia. In this study, all in the control group experienced mild hypothermia, and their mean time of awakening from anesthesia was longer than that of their experimental group peers. In previous studies (Aarnes et al., 2017; Hines et al., 1992; Lenhardt et al., 1997), IPH was found to negatively affect the time of awakening from anesthesia. However, no study has been found in the literature revealing the relationship between IPH and time of awakening from anesthesia. The results of this study contributes to the literature by comparing patients who all received one type of anesthesia (general anesthesia) and were given the same drugs (evidence level = A2). Thus, this finding confirms the validity of hypothesis H1_a_. Although the findings require further confirmation and validation in future studies, the absence of IPH in the experimental group and subsequent faster recovery time from anesthesia support the effectiveness of using the NC.

Actively warming surgical patients is associated with reduced perioperative blood loss through the prevention of IPH-associated coagulopathy. Therefore, perioperative active warming may be applied routinely to all patients scheduled for various surgeries to prevent the negative consequences of IPH (Xiong et al., 2022). Risk of IPH is often overlooked in the clinical setting, as coagulation laboratory studies involving PT and partial thromboplastin time are usually performed at a temperature of 37°C rather than at the patient's actual temperature. When conventional coagulation tests (PT, aPTT, INR) were analyzed under normothermic conditions established in vitro, it was determined that aPTT and PLT count were not affected by mild hypothermia. However, when this analysis was performed at the patient's own BT, a gradual hypocoagulative response was observed (Rohrer & Natale, 1992). This result indicates bleeding tendency increases with decreased patient BT. In a study using experimental and control groups (Zheng et al., 2017), experimental group patients were actively heated using several methods before and after their operation, whereas control group patients were not heated. The PT and aPTT values of the heated patients were significantly different than those of the nonheated patients. Several studies have investigated the effects of mild hypothermia on blood coagulation and PLT function. An experimental study by Wolberg et al. (2004) showed that tendency to bleed increased at temperatures below 33°C. In the study of Shimokawa et al. (2003) on hypothermic patients, hypothermic patients were found to have a significant hypocoagulative response. In this study, all of the patients in the control group experienced mild hypothermia and exhibited a higher tendency to bleed than their experimental group peers. Although no signs of bleeding were found in laboratory tests in this study, the risk of bleeding in the control group was higher than that in the experimental group. These results indicate IPH can cause coagulation disorders even in cases of mild hypothermia and thus pose a significant bleeding risk to patients. In addition, the increases in PT, aPTT, and INR values and decreases in PLT, HCT, Hg, and Ca^+2^ values between presurgery and postsurgery indicate normal functioning of the coagulation cascade is impaired in mild hypothermia. These results confirm hypothesis H1_b_.

### Implications for Practice

IPH is a precursor of many complications in patients. In surgical patients, IPH can be prevented using evidence-based nursing care. Standardizing nursing care to prevent IPH positively affects both quality of care and patient outcomes. The NC guidelines developed in this study provide an effective protocol for preventing IPH that can accelerate the discharge of anesthesia, reduce risk of bleeding, and prevent other complications in patients undergoing surgery. In the future, it is important for nurses to use NC interventions in patient care and for new studies to be conducted that show patient outcomes.

### Conclusions

IPH is a complication that may be prevented using evidence-based practices. Nurses are responsible to achieve and maintain normothermia, interact with anesthesiologists and surgeons, apply passive insulation and active heating methods, lead the surgical team, and control the temperature in the operating room. Proactive and evidence-based nurse engagement in the surgical team can better protect patients from the negative effects of IPH. Applying the NC guidelines in this study helped patients wake up more easily from anesthesia and contributed to the prevention of coagulation disorders that could lead to bleeding. Therefore, the NC guidelines developed in this study for the prevention of IPH were shown to be effective, appropriate, and clinically applicable. In line with these results, more studies in other surgical clinics using the developed NC guidelines and involving other nursing-led IPH preventive interventions should be conducted. In addition, nurses should participate in research and patient care activities aimed at preventing IPH, evaluating IPH using BT measured by different methods, evaluating the efficacy of different active warming techniques on IPH outcomes, and evaluating the efficacy of different anesthetization methods on IPH outcomes.

### Limitations

This study has certain limitations. First, this study used a relatively small sample of patients treated in a general surgery clinic in a single hospital in Turkey. At the same time, this study was carried out during the most intense periods of the COVID-19 pandemic in Turkey. The number of elective surgeries was reduced by 90% in the hospital where the study was conducted. For this reason, the study was terminated when the minimum sample size in the power analysis was reached. The BT values of the participants were measured using a single instrument, as central temperature measurements such as in the esophagus, bladder, and rectum could not be made. The participants were warmed using a forced-air warming device, which is an active heating technique. Although fluid warmers were also used, because other active warming techniques were not used, patients were evaluated using a uniform warming method. All of the patients were anesthetized using an IV.
